# Rho-ROCK signaling mediates entotic cell death in tumor

**DOI:** 10.1038/s41420-020-0238-7

**Published:** 2020-01-23

**Authors:** Chong Zeng, Boning Zeng, Changjiang Dong, Jing Liu, Feiyue Xing

**Affiliations:** 1 Institute of Tissue Transplantation and Immunology, Department of Immunobiology, Jinan University, Guangzhou, China; 2 MOE Key Laboratory of Tumor Molecular Biology, Key Laboratory of Functional Protein Research of Guangdong, Higher Education Institutes, Jinan University, Guangzhou, China; 3 BioMedical Research Centre, University of East Anglia, NR4 7TJ Norwich, UK; 4 School of Stomatology, Jinan University, Guangzhou, China

**Keywords:** RHO signalling, Entosis

Entosis was first described by Overholtzer et al. in 2007. They found a new cell elimination process, in which breast cancer cells enter neighboring cells to form cell-in-cell structures and then the internalized cells are either degraded by lysosomal enzymes or released unlike cannibalistic or phagocytic forms of engulfment^1^. Entosis is a non-apoptotic cell death process, which does not trigger nuclear fragmentation or cleavage of caspase-3^1^. Entosis is induced in human breast cancer cells, which is concerned with epithelial adherens junction (AJ) consisted of epithelial-cadherin (E-cadherin) and AJ/cytoskeleton linker protein α-catenin^2,3^. Given that E-cadherin regulates cell–cell adhesions by homotypic interactions with E-cadherin molecules on neighboring cells,^4^ entosis primarily occurs in sibling cells. The expression of exogenous E-cadherin proteins is adequate to trigger entosis in human tumor cells,^5,6^ indicating that E-cadherin is necessary for the occurrence of entosis. Once cell–cell adhesion forms between the “winner” (engulfing) and the “loser” (entotic) cells, surprisingly, the loser cells zealously invade the winner cells through actomyosin contraction. This process is mediated by the Rho-ROCK signaling pathway^6,7^. Here, we concisely feature a potential main mechanistic process of entosis.

**Fig. 1:**
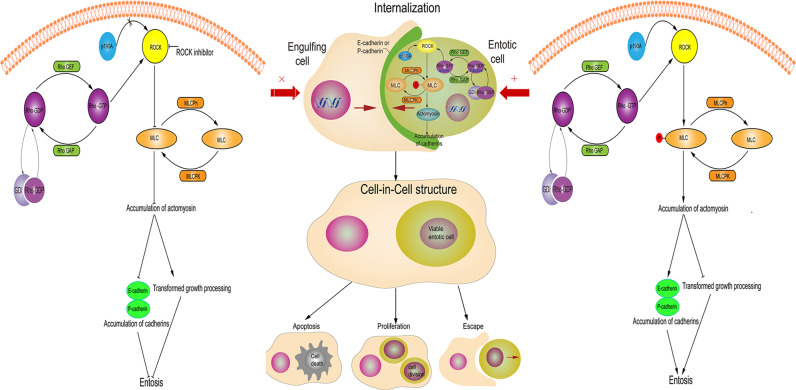
Proposed schematic pathway for the regulation of entosis. While the Rho (Rho-GTP) is activated, sequential activation of Rho-kinase with p190A Rho-GTPase-activating protein (p190A Rho-GAP) occurs and then phosphorylates MLC. As a result, the increased phosphorylation of MLC leads to more accumulation of actomyosin, contributing to the expression of cadherin (E-cadherin or P-cadherin) and suppressing the transformed growth in cells and the induction of entosis. When the Rho (Rho-GTP) is activated, lack of p190A or presence of ROCK inhibitors suppresses the MLC to reduce the actomyosin, resulting in the inhibition of cadherin and promoting transformed growth in cells with the attenuation of entosis.

The Rho-ROCK signaling pathway consists of Rho-GTP family and its downstream effector ROCK. The Rho family of GTPases is a family of small proteins which includes three members, Rho (RhoA, RhoB, and RhoC), Rac (Rac1, Rac2, and Rac3) and cell division cycle 42 (Cdc42), and the ROCK family contains two isoforms, ROCK1 and ROCK2^8^. The Rho GTPases take part in various cell biobehaviours, including cell adhesion, migration, and contraction. As a molecular switch, the Rho GTPases cyclically exchange between active (GTP-bound) and inactive (GDP-bound) conformations. The cycling is controlled by three regulatory proteins, GTPase-activating protein (GAP), guanine dissociation inhibitor (GDI) and guanine-nucleotide-exchange factor (GEF) (Fig. 1)^9^. The activation of the Rho-GTPase is induced by Rho-GEF that exchanges GTP with GDP, and its inactivation is catalyzed by Rho-GAPs. Moreover, GDI can seclude the Rho-GDP in cytosol, protecting it from reactivation triggered by GEF. The Rho-GTPases bind to cell membrane, and then interact with various effector molecules to initiate cellular responses^10,11^. The active Rho-GTP combines its targets, formins and ROCK, to incur the polymerization of actin, the phosphorylation of myosin light chain (MLC) and the suppression of myosin light-chain phosphatase (MLCPh) activity, eventually promoting the forming of actomyosin structures^10,12^.

The vital driving force from engulfed cells can facilitate themselves to be taken up by engulfing cells through actin polymerization and myosin heavy chain-II contraction in a Rho-GTPase-dependent manner^6^. The actin and myosin heavy and light chains are particularly enriched in the “loser” cells at their cortex opposite to the cell–cell junctional interface between the “loser” and “winner” cells, and the resulting mechanical tension generated by the difference of polarized distribution of RhoA activity and contractile actomyosin between both the cells promotes the formation of cell-in-cell structures^5,13^. Furthermore, the Rho-GTPase-activating protein p190A-RhoGAP (p190A), a Rho inactivator, can be recruited to cell–cell adhesions by cadherin. Subsequently, Rho is activated by PDZ-Rho-GEF in the distal cortex of invaded cells (Fig. 1)^6,7^. Thus, a zone of the polarized actomyosin contraction is established, promoting the engulfed cell uptake. The knock down of the p190A reduces cell–cell adhesion, and inhibits entosis of cells^7^. Once ingested, engulfed cells are primarily killed by engulfing cells, but some of them can escape from their hosts, appearing unharmed and undergoing subsequent cell division. Interestingly, internalized cells can also divide within their host cell vacuoles^14^.

Overall, entosis is a non-apoptotic form of “cell-in-cell” structures in tumor. Different from traditional cell death processes, such as apoptosis, pyroptosis, and necrosis, it is involved in live cell invasion into its neighbors with lysosome fusion, internalized cell death and degradation, not triggering nuclear fragmentation or cleavage of caspase-3 or caspase-1/-11 or alteration of rapamycin activity^1^. This cell death is associated with E-cadherin expression, RhoA-GTPase, and ROCK activity within engulfed cells, relying on p190A activity. Its detailed mechanistic process remains confused. The emerging data indicate that the activation of the Rho-ROCK/MLC/cadherin signaling pathway triggers entotic cell death and the ROCK functions as a pivotal switch in the executed entosis. However, why are some entotic cells able to avoid death by escaping from their host? It is relatively important for understanding of tumor occurrence, development, and metastasis to elucidate this physiological and pathological process.
